# Correlation between urinary biomarker and organ failure in patients with sepsis and patients after esophagectomy: a prospective observational study

**DOI:** 10.1186/s40560-020-0428-7

**Published:** 2020-01-17

**Authors:** Chieko Mitaka, Chika Ishibashi, Izumi Kawagoe, Takashi Hashimoto, Makoto Takahashi, Daizoh Satoh, Eiichi Inada

**Affiliations:** 1 Department of Anesthesiology and Pain Medicine, Juntendo University Faculty of Medicine, 3-1-3 Hongo, Bunkyo-ku, Tokyo, 113-8431 Japan; 2 Department of Esophageal and Gastroenterological Surgery, Juntendo University Faculty of Medicine, 3-1-3 Hongo, Bunkyo-ku, Tokyo, 113-8431 Japan; 3 Department of Coloproctological Surgery, Juntendo University Faculty of Medicine, 3-1-3 Hongo, Bunkyo-ku, Tokyo, 113-8431 Japan

**Keywords:** Acute kidney injury, Biomarker, Esophageal cancer, Esophagectomy, NGAL (neutrophil gelatinase-associated lipocalin), Organ dysfunction, Sepsis, Severity

## Abstract

**Background:**

Neutrophil gelatinase-associated lipocalin (NGAL) is a diagnostic marker for acute kidney injury (AKI). NGAL expression is highly induced not only in kidney injury but also in bacterial infection, inflammation, and cancer. The factors regulating NGAL expression are proinflammatory cytokines, and plasma NGAL levels have been increased in septic shock. However, there are no reports of urine neutrophil gelatinase-associated lipocalin (uNGAL) levels after open esophagectomy.

**Methods:**

We prospectively enrolled critically ill patients, including patients with sepsis (*n* = 45) and patients who underwent open esophagectomy (*n* = 40). We compared vital signs, PaO_2_/F_I_O_2_, serum C-reactive protein (CRP) levels, acute physiology and chronic health evaluation (APACHE) II score, sequential organ failure assessment (SOFA) score, and uNGAL levels between the sepsis group and the esophagectomy group. Then, we investigated whether uNGAL is associated with the severity of illness and organ failure, and whether uNGAL is a reliable screening test for AKI.

**Results:**

The median uNGAL levels, APACHE II score, SOFA score, and serum CRP levels were significantly (*p* < 0.001) higher in the sepsis group than in the esophagectomy group on ICU day 1. In the sepsis group, uNGAL levels were significantly (*p* < 0.05) correlated with APACHE II score and SOFA score on intensive care unit (ICU) day 1, 2, and 3. In the esophagectomy group, uNGAL levels were significantly (*p* < 0.05) correlated with SOFA score on ICU day 3 and 4. In the sepsis group, 1 patient developed AKI stage 2 and 6 patients developed AKI stage 3. No patients developed AKI in the esophagectomy group. In a total of 85 patients of this study, 80 patients had an abnormal value of uNGAL and only 7 patients (8.7%) of those 80 patients developed AKI.

**Conclusions:**

uNGAL levels were correlated with the severity of illness and organ failure in critically ill patients. The value of uNGAL increases under the surgical and inflammatory responses, thereby losing a significance of a screening test of AKI in critically ill patients.

## Background

Neutrophil gelatinase-associated lipocalin (NGAL), a 25 kDa protein of the lipocalin family, is a diagnostic marker for acute kidney injury (AKI) [1–3]. NGAL expression is highly induced not only in kidney injury [4, 5] but also bacterial infection, inflammation, and cancer [6–9]. The factors regulating NGAL expression are proinflammatory cytokines such as interleukins, tumor necrosis factor-α and interferons [7]. In fact, plasma NGAL levels have been increased in patients with septic shock [10–12].

On the other hand, open esophagectomy for esophageal cancer performed through a right-thoracotomy and laparotomy is a major invasive surgery [13, 14]. Surgical stress of radical esophagectomy induces the release of interleukin-6 and interleukin-8 and the overproduction of these cytokines induces systemic inflammatory response syndrome [15]. Therefore, open esophagectomy has a higher risk of intraoperative and postoperative complications. Although NGAL is highly expressed in esophageal squamous cell carcinoma [8, 9], there are no reports of uNGAL levels after open esophagectomy. In addition, NGAL is released from the lung, bronchi, and esophagus [7]. Therefore, we surmised that NGAL might be released from various organs such as the lung, bronchi, and esophagus during and after esophagectomy. The design of the present study required the selection of patients at risk for organ dysfunction. Therefore, we prospectively recruited critically ill patients with sepsis and patients after open esophagectomy with gastric reconstruction for esophageal cancer. Accordingly, we investigated in critically ill patients whether uNGAL is associated with the severity of illness and organ failure, and whether uNGAL is a reliable screening test for AKI.

## Methods

### Study design and patients

This CUBIC (correlation between urinary biomarker and organ failure in critically ill patients) study was a prospective observational study. The study protocol was approved by the Ethics Committee of Juntendo University Hospital. This study was performed in accordance with the ethical standards laid down in the 1964 Declaration of Helsinki and its later amendments. Informed written consent was obtained from patients or close relatives. This study was registered with the University Hospital Medical Information Network (UMIN 000024155). From January 2017 to April 2019, we prospectively enrolled 85 critically ill patients who were admitted to the intensive care unit (ICU) at the Juntendo University Hospital. Patients were followed up for 90 days after enrollment. The inclusion criteria were age ≥ 20 years, critically ill patients with either sepsis or open esophagectomy with gastric reconstruction for esophageal cancer. Sepsis was defined as an increase in sequential organ failure assessment (SOFA) score [16] ≥ 2 points caused by a dysregulated host response to infection according to definitions for Sepsis-3 [17]. The exclusion criteria were end-stage kidney disease and renal replacement therapy prior to intensive care unit (ICU) admission or kidney transplant. End-stage kidney disease is defined by a need for dialysis longer than 3 months [18].

### Data collection

Demographic data for each participant were collected, including age, gender, and underlying diseases. The vital signs and arterial blood gas of each patient were measured and recorded. Routine blood tests including C-reactive protein (CRP) level were conducted in the central laboratory of the hospital, and the results of the tests were recorded. uNGAL and urine creatinine levels were measured on ICU day 1, 2, 3, and 4. Spot urine was taken at admission to the ICU on day 1 and at 9:00 am on ICU day 2, 3, and 4. The definition of AKI was based on the Kidney Disease: Improving Global Outcomes (KDIGO) criteria: an increase in serum creatinine of at least 0.3 mg/dL from the baseline level within 48 h (AKI stage 1), serum creatinine 2.0–2.9 times baseline (AKI stage 2) and serum creatinine 3.0 times baseline or increase in serum creatinine ≥ 4.0 mg/dL (AKI stage 3) [19]. Acute physiology and chronic health evaluation (APACHE) II score [20] and SOFA score [16] were calculated.

Urine samples for uNGAL measurement were centrifuged at 1500 g for 10 min and the supernatants were stored at − 80 °C. uNGAL was measured by a chemiluminescent microparticle immunoassay using the ARCHITECT analyzer i2000 SR (Abbott Japan Co., Ltd., Tokyo, Japan). uNGAL was expressed as ng/mg creatinine to standardize and correct for changes in urine concentration. The upper limit level of uNGAL in a healthy subject was 30.5 ng/mL or 21.7 ng/mg creatinine.

In the sepsis group, crystalloid solution (30 mL/kg or more) was administered as initial fluid resuscitation at the early phase of sepsis, and 5% albumin was administered in patients with hypoalbuminemia. Noradrenalin and vasopressin were administered to maintain mean arterial pressure more than 65 mmHg. Dobutamine was administered in patients with cardiac dysfunction. In the esophagectomy group, fluid infusion was controlled by hemodynamic monitoring and gradually reduced from ICU day 3. Mechanical ventilation was used in patients with respiratory failure in both groups.

We investigated the relationships between uNGAL levels and the severity of illness, organ failure, and inflammation in critically ill patients. In addition, we calculated the sensitivity and specificity of uNGAL for diagnosing AKI in a total of 85 patients.

### Statistical analysis

Quantitative data are expressed as the median and interquartile range (IQR). The intergroup differences were compared using the Mann-Whitney *U* test. Categorical data are expressed as absolute values and percentages, and were analyzed using the chi-square test. The association of uNGAL levels with APACHE II score, SOFA score, and CRP levels were evaluated by Spearman’s rank correlation test. *P* < 0.05 was considered statistically significant.

## Results

### Patient characteristics

Critically ill patients included patients with sepsis (*n* = 45) and patients after open esophagectomy with gastric reconstruction for esophageal cancer (*n* = 40). Sources of sepsis were intra-abdominal (*n* = 31), urinary tract (*n* = 8), bloodstream (*n* = 2), and immunosuppression (*n* = 4) in patients with sepsis.

### Comparison of various parameters between the sepsis group and the esophagectomy group

A comparison of various parameters between the sepsis group and the esophagectomy group on ICU day 1, 2, 3, and 4 are shown in Table 1. Although there was no significant difference in age between the two groups, the male ratio was significantly (*p* = 0.0078) lower in the sepsis group than in the esophagectomy group. The median body temperature was significantly (*p* = 0.0092) lower in the sepsis group than in the esophagectomy group on ICU day1. However, the median body temperature was significantly (*p* = 0.0381) higher in the sepsis group than in the esophagectomy group on ICU day 4. The median mean arterial pressure was significantly (*p* = 0.0047) lower in the sepsis group than in the esophagectomy group on ICU day 1. The median PaO_2_/F_I_O_2_ ratio was significantly (*p* < 0.05) higher in the sepsis group than in the esophagectomy group on ICU day 1, 2, 3, and 4. On the other hand, the median serum creatinine level was significantly (*p* = 0.0006) higher in the sepsis group than in the esophagectomy group on ICU day 1. The median fluid infusion was significantly (*p* = 0.0204) higher in the sepsis group than in the esophagectomy group on ICU day 1. The median uNGAL levels were significantly (*p* < 0.001) higher in the sepsis group than in the esophagectomy group on ICU day 1, 2, 3 and 4. The median APACHE II score and median CRP level were significantly (*p* < 0.001) higher in the sepsis group than in the esophagectomy group on ICU day 1. The median SOFA score was significantly (*p* < 0.05) higher in the sepsis group than in the esophagectomy on ICU day 1 and 2.

**Table 1:** Comparison of various parameters between the sepsis group and the esophagectomy group

	All (*n* = 85)	Sepsis group (*n* = 45)	Esophagectomy group (*n* = 40)	*P* value
Age, years	67 [59–75]	70 [56–76]	66 [62–72]	0.9470
Male, *n* (%)	51 (60%)	21 (47%)	30 (75%)	0.0078
Body Temperature (°C)				
Day 1	37.1 [36.7–37.9]	36.9 [36.5–37.5]	37.4 [36.9–38.1]	0.0092
Day 2	37.2 [36.8–37.7]	37.1 [36.7–37.6]	37.3 [36.9–37.8]	0.0926
Day 3	37.3 [36.9–37.8]	37.3 [36.8–37.8]	37.3 [37.1–37.8]	0.5998
Day4	37.0 [36.6–37.4]	37.1 [36.9–37.4]	36.8 [36.5–37.2]	0.0381
MAP (mmHg)				
Day 1	71 [61–84]	65 [58–75]	79 [68–93]	0.0047
Day 2	76 [68–86]	76 [70–86]	76 [68–88]	0.8020
Day 3	88 [75–94]	85 [74–94]	89 [76–94]	0.8903
Day 4	90 [74–99]	89 [71–102]	88 [77–95]	0.8111
Heart rate (/min)				
Day 1	93 [85–108]	93 [83–112]	94 [87–106]	0.8395
Day 2	89 [78–99]	86 [74–100]	90 [86–95]	0.4170
Day 3	91 [77–103]	89 [72–106]	92 [87–101]	0.4884
Day 4	89 [74–98]	88 [74–101]	86 [78–101]	0.9632
Respiratory rate (/min)				
Day 1	22 [20–26]	22 [20–26]	23 [20–26]	0.9368
Day 2	20 [18–24]	20 [18–24]	20 [20–23]	0.5720
Day 3	20 [17–24]	19 [16–24]	21 [18–24]	0.1031
Day 4	20 [18–23]	21 [18–23]	20 [19–23]	0.6380
PaO_2_/F_I_O_2_				
Day 1	277 [214–348]	298 [230–412]	243 [203–287]	0.0140
Day 2	288 [223–353]	331 [235–444]	260 [212–308]	0.0206
Day 3	258 [210–318]	305 [229–346]	231 [207–289]	0.0101
Day 4	257 [210–315]	320 [264–353]	240 [187–269]	< 0.0001
Fluid (mL/h)				
Day 1	132 [110–180]	165 [118–229]	124 [108–151]	0.0204
Day 2	125 [106–148]	134 [103–167]	120 [107–132]	0.1036
Day 3	111 [100–132]	110 [95–150]	110 [100–125]	0.7830
Day 4	98 [89–113]	103 [89–132]	96 [87–104]	0.0736
sCr (mg/dL)				
Day 1	0.80 [0.59–1.16]	1.11 [0.67–1.92]	0.72 [0.57–0.82]	0.0006
Day 2	0.82 [0.59–1.08]	0.9 [0.61–1.33]	0.76 [0.59–0.90]	0.0978
Day 3	0.72 [0.59–1.02]	0.75 [0.6–1.26]	0.69 [0.53–0.95]	0.2986
Day 4	0.63 [0.44–0.86]	0.70 [0.5–1.23]	0.61 [0.44–0.76]	0.1602
eGFR (mL/min/1.73 m^2^)				
Day 1	72 [44–93]	46 [27–85]	82 [71–97]	< 0.0001
Day 2	66 [47–92]	57 [37–85]	75 [64–94]	0.0137
Day 3	74 [54–93]	69 [38–87]	85 [62–99]	0.0501
Day 4	86 [67–114]	78 [39–98]	98 [74–118]	0.0214
uNGAL (ng/mg creatinine)				
Day 1	81.1 [26.2–461]	461.4 [179.5–1893.9]	27.1 [16.0–64.9]	< 0.0001
Day 2	66.4 [21.3–311.2]	310.8 [108.4–1559.0]	20.6 [11.9–37.7]	< 0.0001
Day 3	52.4 [26.0–198.1]	211.5 [93.4–889.9]	29.4 [16.5–44.5]	< 0.0001
Day 4	43.8 [26.9–100.5]	217.7 [39.4–1285.8]	37.9 [22.1–64.6]	0.0002
CRP (mg/dL)				
Day 1	1.7 [0.6–12.2]	12.1 [5.4–19.2]	0.6 [0.3–0.8]	< 0.0001
Day 2	12.0 [8.6–19.6]	19.0 [10.5–23.7]	9.7 [8.3–12.0]	< 0.0001
Day 3	18.5 [12.6–23.8]	18.2 [12.5–25.9]	18.8 [14.1–23.6]	0.9544
Day 4	14.3 [11.1–19.6]	13.4 [11.2–16.0]	14.8 [11–20.4]	0.5600
APACHE II score	12 [9–14]	14 [10–18]	10 [8–12]	< 0.0001
SOFA score				
Day 1	4 [2–7]	6 [2–10]	3 [2–4.3]	0.0006
Day 2	3 [2–5]	4 [2–6]	2 [2–3]	0.0323
Day 3	3 [2–4]	3.5 [3–5.8]	3 [2–5.5]	0.8503
Day 4	3 [2–5]	4 [2–5]	3 [2–4]	0.5415
ICU stay (days)	8 [5–8]	6 [3–10]	8 [8–8]	0.0043

The qualitative data are shown as number (percentage) and the quantitative data are shown as medians [interquartile ranges]. APACHE II, acute physiology and chronic health evaluation II; CRP, C-reactive protein: eGFR, estimated glomerular filtration rate; ICU, intensive care unit; MAP, mean arterial pressure; SOFA, sequential organ failure assessment; sCr, serum creatinine; uNGAL, urine neutrophil gelatinase-associated lipocalin

*P* value, sepsis vs. esophagectomy

In the sepsis group, a combination of noradrenalin and vasopressin were administered in 6 patients and noradrenalin was administered in 18 patients, and dobutamine was administered in 3 patients. In the sepsis group, 7 patients underwent mechanical ventilation and the median duration of mechanical ventilation was 7 [IQR 4–17] days. In the esophagectomy group, 2 patients underwent mechanical ventilation and the duration of mechanical ventilation was 115 days and 18 days, respectively.

### Relationships between uNGAL and APACHE II score, SOFA score, and CRP

**Fig. 1:**
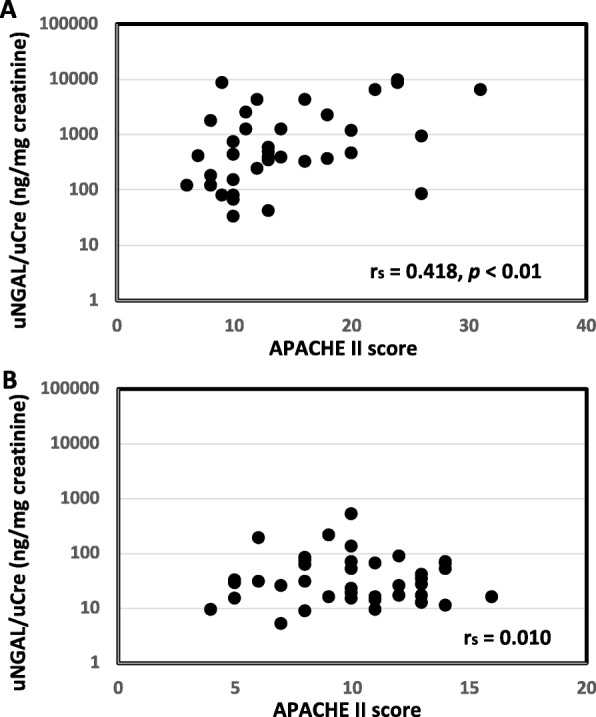
Relationship between urine neutrophil gelatinase-associated lipocalin (uNGAL) level and acute physiology and chronic health evaluation (APACHE) II score in the sepsis group (A) and the esophagectomy group (B). *r*_s_: correlation coefficient calculated from Spearman’s rank correlation test

**Fig. 2:**
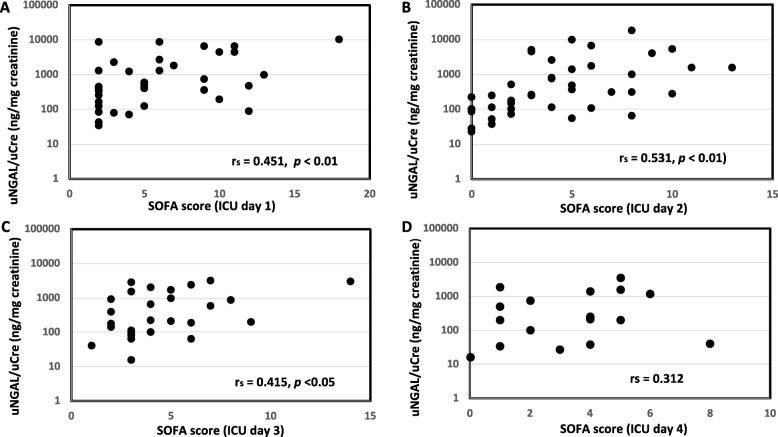
Relationship between urine neutrophil gelatinase-associated lipocalin (uNGAL) level and sequential organ failure assessment (SOFA) score in the sepsis group on ICU day 1 (A), day 2 (B), day 3 (C), and day 4 (D)

**Fig. 3:**
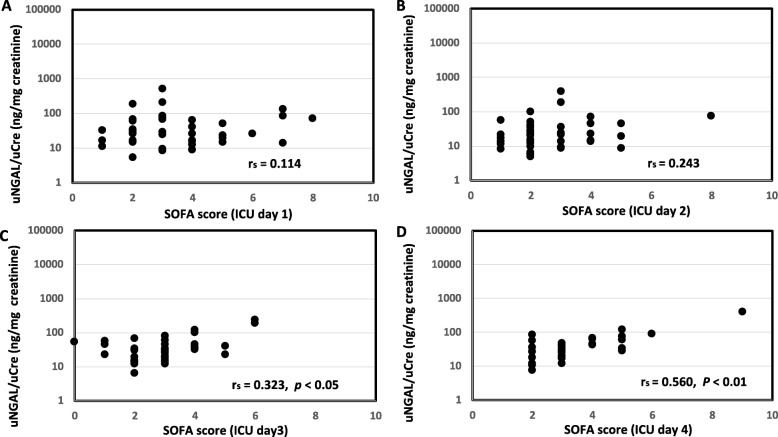
Relationship between urine neutrophil gelatinase-associated lipocalin (uNGAL) level and sequential organ failure assessment (SOFA) score in the esophagectomy group on ICU day 1 (A), day 2 (B), day 3 (C), and day 4 (D). *r*_s_: correlation coefficient calculated from Spearman’s rank correlation test

uNGAL levels were significantly correlated with APACHE II score in the sepsis group (*r*_s_ = 0.418, *p* < 0.01, Fig. 1A), but uNGAL levels were not significantly correlated with APACHE II score in the esophagectomy group (*r*_s_ = 0.011, Fig. 1B). In the sepsis group, uNGAL levels were significantly correlated with SOFA score on ICU day 1 (*r*_s_ = 0.451, *p* < 0.01, Fig. 2A), day 2 (*r*_s_ = 0.531, *p* < 0.01, Fig. 2B), day 3 (*r*_s_ = 0.415, *p* < 0.05, Fig. 2C), but not on day 4 (*r*_s_ = 0.312, Fig. 2D). In the esophagectomy group, uNGAL levels were not significantly correlated with SOFA score on ICU day 1 (*r*_s_ = 0.114, Fig. 3A) and day 2 (*r*_s_ = 0.243, Fig. 3B), but they were significantly correlated with SOFA score on day 3 (*r*_s_ = 0.323, *p* < 0.05, Fig. 3C) and day 4 (*r*_s_ = 0.560, *p* < 0.01, Fig. 3D). In the sepsis group, uNGAL levels were not significantly correlated with serum CRP levels on ICU day 1 (*r*_s_ = 0.114), day 2 (*r*_s_ = 0.077), and day 3 (*r*_s_ = 0.262). In the esophagectomy group, uNGAL levels were not significantly correlated with serum CRP levels on ICU day 1 (*r*_s_ = 0.008), day 2 (*r*_s_ = 0.114), and day 3 (*r*_s_ = 0.05).

### Relationship between uNGAL and AKI

uNGAL levels were > 21.7 ng/mg creatinine (above normal range) in all septic patients. The details of uNGAL level in septic patients were > 21.7 to < 50 ng/mg creatinine (*n* = 2), 50 to < 150 ng/mg creatinine (*n* = 11), 15 to < 1000 ng/mg creatinine (*n* = 17), and > 1000 ng/mg creatinine (*n* = 15). In the sepsis group, one patient developed AKI stage 2 and 6 patients developed AKI stage 3, who underwent continuous renal replacement therapy. The median uNGAL level in patients with AKI stage 3 was 2582.6 [IQR 1780–4457.8] ng/mg creatinine on ICU day 1. The median uNGAL level was 3520.2 [IQR 1980.7–5932.5] ng/mg creatinine in 7 septic patients with AKI on ICU day 1. Among these patients, uNGAL levels were remarkably elevated in 1 patient with loops enteritis (18,473.5 ng/mg creatinine) on ICU day 2 and 1 patient with obstructive jaundice due to cholangiocarcinoma (10,069.1 ng/mg creatinine) (AKI stage 2) on ICU day 1. On the other hand, the peak uNGAL levels of 8 patients with urinary tract infection were 5203.7, 354.7, 3979.9, 10011, 6799.4, 4457.8, 4268.9, and 8660 ng/mg creatinine. Only 1 of these patients developed AKI stage 3 whose uNGAL level was 4457.8 ng/mg creatinine and underwent continuous renal replacement therapy. In the esophagectomy group, uNGAL levels were < 21.7 ng/mg creatinine (within normal range) (*n* = 5), 21.7 - < 50 ng/mg creatinine (*n* = 11), 50 - < 150 ng/mg creatinine (*n* = 19), and 150 - < 1000 ng/mg creatinine (n = 5). No patients after esophagectomy developed AKI.

In a total of 85 patients of this study, 80 patients had an abnormal value of uNGAL and only 7 patients (8.7%) of those 80 patients developed AKI. At a cutoff value of 50 ng/mg creatinine, sensitivity and specificity of uNGAL for detecting AKI were 1.0 and 0.231, respectively. At a cutoff value of 150 ng/mg creatinine, the sensitivity and specificity of uNGAL for detecting AKI were 1.0 and 0.615, respectively.

uNGAL levels were not correlated with serum creatinine levels or eGFR in critically ill patients on each ICU day. Five patients with sepsis died within 90 days. The peak uNGAL levels of the 5 patients who died were 2889, 66, 1540, 10011, and 471 ng/mg creatinine.

## Discussion

The major findings in the present study were that uNGAL levels were correlated with the severity of illness, organ failure, and inflammation in critically ill patients. In a total of 85 patients of this study, 80 patients had an abnormal value of uNGAL and only 7 patients (8.7%) of those 80 patients developed AKI. The positive predictive value of AKI was 0.088. Accordingly, a diagnostic ability of uNGAL for AKI can be masked under the surgical and inflammatory responses, thereby losing a significance of a screening test of AKI.

### Relationship between uNGAL and APACHE II score, SOFA score, and CRP

The present study showed that uNGAL levels were significantly correlated with APACHE II score in the sepsis group. These findings indicate that uNGAL levels were correlated with the severity of illness in the sepsis group. In addition, the present study showed that uNGAL levels were significantly correlated with SOFA score in the sepsis group on ICU days 1, 2, and 3. On the other hand, uNGAL levels were significantly correlated with SOFA score on ICU day 3 and 4 in the esophagectomy group. These findings indicate that uNGAL levels were associated with organ failure, although the pattern of the sepsis group and the esophagectomy group was different. In the sepsis group, the relationships between uNGAL and APACHE II score, SOFA scores were found on ICU day 1, 2, and 3, which were not found in the esophagectomy group. These results mean the increase in uNGAL levels was remarkable and occurred in the early phase of sepsis. Wang et al. [10] showed that high plasma NGAL levels were significantly associated with the APACHE II score, the SOFA score, and the CRP levels in severe sepsis and septic shock. Their study only measured plasma NGAL levels to assess the correlation with severity and organ failure. Although our study measured not plasma NGAL but uNGAL levels, relationships between uNGAL and APACHE II score and SOFA score are similar to their study. On the other hand, including patients after esophagectomy is different from their study. Shapiro NI, et al. [12] have reported in a multicenter observational study that a combination of plasma NGAL, interleukin-1 receptor antagonist, and protein C was predictive of organ dysfunction in 971 patients with suspected sepsis presenting to the emergency department. Because their study included not only plasma NGAL but also interleukin-1 receptor antagonist and protein C as a predictor of organ dysfunction, their study is different from our study.

### Relationship between uNGAL and AKI

In the present study, uNGAL levels were 50 to < 150 ng/mg creatinine (*n* = 30), 150 to < 1000 ng/mg creatinine (*n* = 28), and > 1000 ng/mg creatinine (*n* = 15). de Geus et al. [21] have reported cardiac surgery-associated NGAL score as a potential tool to monitor acute tubular damage. This score shows that uNGAL level 50 to < 150 ng/mL is tubular damage possible, uNGAL level 150 to < 1000 ng/mL is tubular damage, and uNGAL level > 1000 ng/mL is severe tubular damage. The absolute cutoff level of 150 ng/mL for tubular damage was derived from identifying patients with acute tubular damage related to cardiac surgery or critical illness. Ueta K, et al. [22] demonstrated that the cutoff level of uNGAL for AKI prediction was 65.1 ng/mg creatinine in patients after endovascular stent graft repair of aortic aneurysm. Considering these findings, the present study showed that more than half of the patients had risk of tubular damage.

The median uNGAL level was significantly elevated to 3520.2 ng/mg creatinine in 7 septic patients with AKI on ICU day 1, and 6 of these patients underwent continuous renal replacement therapy. Interestingly, 7 out of 8 patients with urinary tract infections, whose uNGAL levels were extremely elevated, did not develop AKI. Although Mori et al. [23] proposed that NGAL expression is a real-time indicator of active renal injury, we could not demonstrate an ability of uNGAL for predicting AKI in critically ill patients in the present study. Furthermore, although de Geus et al. [24] demonstrated that plasma NGAL and uNGAL levels at the time of ICU admission predict the development of severe AKI and the initiation of renal replacement therapy in critically ill patients, the results of the present study did not support the usefulness of uNGAL for predicting AKI.

Shavit et al. [25] evaluated serum NGAL and uNGAL as a predictor of AKI, morbidity and mortality in patients who underwent non-cardiac major surgery. No significant correlation was detected between serum NGAL or uNGAL and subsequent development of AKI. None of their patients developed severe AKI or required renal replacement therapy, which may reduce the predictive capacity of NGAL for AKI. However, serum NGAL or uNGAL was strongly correlated with postoperative infection and death. Their finding that none of their patients developed severe AKI or required renal replacement therapy is compatible with our patients after esophagectomy. In addition, their finding that surgical and inflammatory response and infection may reduce the predictive capacity of NGAL is very similar to our study.

### Study limitation

There are several limitations in the present study. First, this study is a single-center study with a small number of critically ill patients. Second, we only measured uNGAL levels and did not measure plasma NGAL levels. Plasma NGAL is a marker of systemic inflammatory conditions, whereas uNGAL is specific for injury to the renal epithelium [26]. Therefore, uNGAL levels in patients with urinary tract infection were remarkably elevated in the present study. uNGAL levels and plasma NGAL levels may represent a more detailed situation in sepsis.

## Conclusions

In conclusion, our results indicate that uNGAL levels were correlated with the severity of illness and organ failure in critically ill patients. A diagnostic ability of uNGAL for AKI can be masked under the surgical and inflammatory responses, thereby losing a significance of a screening test of AKI. The uNGAL level in patients with sepsis and patients after major surgery must be interpreted carefully by considering the inflammatory response and organ failure of the patients. Further large-scale studies are needed to investigate the role of NGAL in sepsis.

## Data Availability

The datasets analyzed during the current study are available from the corresponding author on reasonable request.

## Abbreviations

AKI: Acute kidney injury
APACHE: Acute physiology and chronic health evaluation
CRP: C-reactive protein
ICU: Intensive care unit
IQR: Interquartile range
KDIGO: Kidney Disease: Improving Outcomes
NGAL: Neutrophil gelatinase-associated lipocalin
sCr: Serum creatinine
SOFA: Sequential organ failure assessment
uCr: Urine creatinine
UMIN: University Hospital Medical Information
uNGAL: Urine neutrophil gelatinase-associated lipocalin
